# Control of ovule development in *Vitis vinifera* by *VvMADS28* and interacting genes

**DOI:** 10.1093/hr/uhad070

**Published:** 2023-04-13

**Authors:** Songlin Zhang, Li Wang, Jin Yao, Na Wu, Bilal Ahmad, Steve van Nocker, Jiuyun Wu, Riziwangguli Abudureheman, Zhi Li, Xiping Wang

**Affiliations:** State Key Laboratory of Crop Stress Biology in Arid Areas, College of Horticulture, Northwest A&F University, Yangling, Shaanxi 712100, China; Key Laboratory of Horticultural Plant Biology and Germplasm Innovation in Northwest China, Ministry of Agriculture, Northwest A&F University, Yangling, Shaanxi 712100, China; State Key Laboratory of Crop Stress Biology in Arid Areas, College of Horticulture, Northwest A&F University, Yangling, Shaanxi 712100, China; College of Horticulture, Hebei Agricultural University, Baoding 071000, China; State Key Laboratory of Crop Stress Biology in Arid Areas, College of Horticulture, Northwest A&F University, Yangling, Shaanxi 712100, China; Key Laboratory of Horticultural Plant Biology and Germplasm Innovation in Northwest China, Ministry of Agriculture, Northwest A&F University, Yangling, Shaanxi 712100, China; State Key Laboratory of Crop Stress Biology in Arid Areas, College of Horticulture, Northwest A&F University, Yangling, Shaanxi 712100, China; Key Laboratory of Horticultural Plant Biology and Germplasm Innovation in Northwest China, Ministry of Agriculture, Northwest A&F University, Yangling, Shaanxi 712100, China; State Key Laboratory of Crop Stress Biology in Arid Areas, College of Horticulture, Northwest A&F University, Yangling, Shaanxi 712100, China; Agriculture Genomics Institute, Chinese Academy of Agricultural Sciences, Shenzhen 518000, China; Department of Horticulture, Michigan State University, East Lansing, MI 48823, USA; Turpan Research Institute of Agricultural Sciences, Xinjiang Academy of Agricultural Sciences, Turpan 838000, Xinjiang, China; Turpan Research Institute of Agricultural Sciences, Xinjiang Academy of Agricultural Sciences, Turpan 838000, Xinjiang, China; State Key Laboratory of Crop Stress Biology in Arid Areas, College of Horticulture, Northwest A&F University, Yangling, Shaanxi 712100, China; Key Laboratory of Horticultural Plant Biology and Germplasm Innovation in Northwest China, Ministry of Agriculture, Northwest A&F University, Yangling, Shaanxi 712100, China; State Key Laboratory of Crop Stress Biology in Arid Areas, College of Horticulture, Northwest A&F University, Yangling, Shaanxi 712100, China; Key Laboratory of Horticultural Plant Biology and Germplasm Innovation in Northwest China, Ministry of Agriculture, Northwest A&F University, Yangling, Shaanxi 712100, China; Turpan Research Institute of Agricultural Sciences, Xinjiang Academy of Agricultural Sciences, Turpan 838000, Xinjiang, China

## Abstract

Seedless grapes are increasingly popular throughout the world, and the development of seedless varieties is a major breeding goal. In this study, we demonstrate an essential role for the grapevine MADS-box gene *VvMADS28* in morphogenesis of the ovule. We found that *VvMADS28* mRNA accumulated in the ovules of a seeded cultivar, ‘Red Globe’, throughout the course of ovule and seed development, especially within the integument/seed coat. In contrast, in the seedless cultivar ‘Thompson Seedless’, *VvMADS28* was expressed only weakly in ovules, and this was associated with increased levels of histone H3 lysine 27 trimethylation (H3K27me3) within the *VvMADS28* promoter region*.* RNAi-mediated transient suppression of *VvMADS28* expression in ‘Red Globe’ led to reduced seed size associated with inhibition of episperm and endosperm cell development. Heterologous overexpression of *VvMADS28* in transgenic tomatoes interfered with sepal development and resulted in smaller fruit but did not obviously affect seed size. Assays in yeast cells showed that *VvMADS28* is subject to regulation by the transcription factor *VvERF98*, and that VvMADS28 could interact with the Type I/ Mβ MADS-domain protein VvMADS5. Moreover, through DNA-affinity purification-sequencing (DAP-seq), we found that VvMADS28 protein specifically binds to the promoter of the grapevine *WUSCHEL* (*VvWUS*) gene, suggesting that maintenance of the VvMADS28–VvMADS5 dimer and *VvWUS* expression homeostasis influences seed development. Taken together, our results provide insight into regulatory mechanisms of ovule and seed development associated with *VvMADS28*.

## Introduction

Table grapes are a nutritious fruit popular with consumers throughout the world. Recently, seedless table grapes have become a focus of marketing, and breeding for seedlessness has become an important goal. In angiosperms, a mature seed comprises the embryo, the endosperm, and the seed coat. The embryo and endosperm originate from the fertilized egg cell and central cell, respectively, while the seed coat develops from the sporophytic integuments [1]. Seedlessness can arise though arrest of embryo development resulting from a variety of conditions, including abnormalities in the micropyle, integument, endosperm or embryo sac, and poor pollination or fertilization [2]. In *Arabidopsis thaliana* (arabidopsis) and rice, several signaling pathways have been shown to control ovule development by influencing the growth of maternal tissues. These include or involve the ubiquitin–proteasome pathway, phytohormone perception, G-protein signaling, and transcriptional regulators [3]. For example, the HAIKU (IKU) pathway controls endosperm development in response to abscisic acid and brassinosteroid signaling, and cytokinin acts downstream of the IKU pathway to control seed growth [4, 5]. Interestingly, some studies have shown that the regulators of seed development are conserved among plant species. For example, three homologous genes from diverse plants have been shown to have similar function: arabidopsis (*STK*) [6], grapevine (*VvAGL11*) [7], and rice (*OsMADS13*) [8]. Therefore, knowledge of mechanisms of seed development in a variety of plants should facilitate understanding of ovule development in grapevine.

The MADS-box gene class has been greatly expanded in higher plants and encodes transcription factors recruited for various roles in development. The studies of MADS-box gene function in plants first focused on floral organ development [9]. MADS-domain proteins interact as homodimers or heterodimers, which expand functional diversity. Recently, several grapevine MADS-box genes involved in seed formation have been discovered. The *VviAGL11* gene plays a key role, and an R197L mutation within the *VviAGL11* open reading frame is associated with the seedless fruit trait [10]; in *VviAGL11-*R197L genotypes, the lignification of endopleura in seeds cannot be initiated, eventually leading to arrested seed development [7]. Functional analyses of additional MADS-box genes, *VvAGAMOUS2* (*VvAG2*), *VvSEPALLATA3* (*VvSEP3)*, *VvMADS39*, and *VvMADS45*, have shown that these four genes also influence seed development [11–13].

*VvMADS28* is a homolog of arabidopsis *FRUITFULL* (*FUL*). *FUL* is required for the development of the fruit valves after fertilization, and transgenic overexpression of *FUL* leads to indehiscent fruit [14]. In *ful* loss of function mutants, siliques are shaped abnormally and the dehiscence zone is not specified [15]. Tomato (*Solanum lycopersic*um) *TDR4*/*FUL1* and *MBP7*/*FUL2*, which have high sequence similarity to arabidopsis *FUL* and appear to predominantly regulate cellular differentiation and fruit ripening [16], can heterodimerize with the MADS-domain ripening regulator RIN [17, 18]. In addition, a *FUL* homolog in bilberry (*Vaccinium myrtillus*) was found to regulate anthocyanin biosynthesis and color development during ripening of the berry [19]. These results indicate that the *FUL* regulatory networks involved in the development of both dry and fleshy fruits may be similar, although the outcomes are morphologically very different. The fruit is a specialized structure that provides a suitable environment for seed maturation and a mechanism for dispersal. In *ful-1* arabidopsis, the lack of coordinated growth of the fruit tissues crowds the seeds and limits seed growth [20]. Fertilization-generated signals may be recognized in the integuments and lead to seed coat differentiation, including activation of MADS-box genes with seed coat-promotive roles. The expression of tomato *TDR4* genes in ovules also supports this [21].

In this study, we focused on the function of the *VvMADS28* gene in ovule development. Expression studies show a defined spatial and temporal expression pattern for *VvMADS28*, suggesting an important role in the establishment of seed identity. Combined with analysis of *VvMADS28* transgenic lines and interactions between proteins, our results suggest a potential function and molecular mechanism of *VvMADS28* in seed development in grapevine.

## Results

### Spatio-temporal expression of *VvMADS28* in grapevine

**Figure 1:**
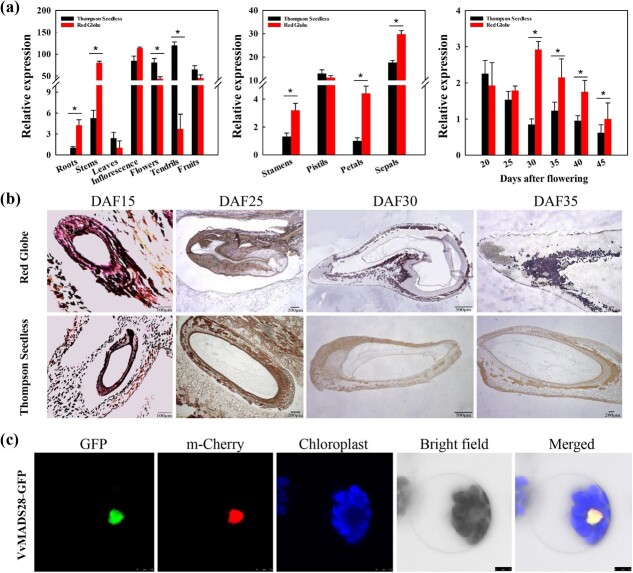
Expression analysis of *VvMADS28* and subcellular localization. **a** Expression pattern of *VvMADS28* in grapevine structures, floral organs, and ovule at progressive developmental stages in ‘Red Globe’ and ‘Thompson Seedless’. Values are means ± standard deviation of three biological replicates; *P < .05 (one-way ANOVA). **b***In situ* hybridization of *VvMADS28* in ‘Red Globe’ and ‘Thompson Seedless’ during ovule developmental. **c** Subcellular localization of VvMADS28-GFP protein expressed in arabidopsis protoplasts. Scale bars = 75 μm.

To gain insight into the potential developmental function of *VvMADS28* in grapevine, we first assessed the expression of *VvMADS28* mRNA in various structures from mature ‘Red Globe’ and ‘Thompson Seedless’ plants. In these two cultivars, *VvMADS28* was found to be expressed in all the analyzed structures, suggesting that it may have a general developmental function (Fig. 1a). For both cultivars, the strongest expression was seen in the inflorescence, flower, and fruit, whereas the weakest expression was seen in the root and leaf. We also assessed expression within the four major floral organs and found that *VvMADS28* was expressed in all of them, with highest expression in the sepal. Finally, we examined expression in ovules at progressive stages of development, from 20 to 45 days after flowering (DAF). During this period, overall expression generally decreased in both cultivars. However, for ‘Red Globe’, strongest expression was seen at 30 DAF, and expression was stronger in ‘Red Globe’ than in ‘Thompson Seedless’ at 30 DAF and later (Fig. 1a). The observed stronger expression in the seeded variety during this period is consistent with a role for *VvMADS28* in seed development.

Moreover, *in situ* hybridization was carried out to document the spatial expression pattern of *VvMADS28* within the developing ovule. In both cultivars, *VvMADS28* mRNA was detected in developing ovules at 15 DAF (Fig. 1b). For ‘Red Globe’, relatively strong expression was seen in the integument at both 30 and 35 DAF. In contrast, almost no expression was observed in ovules of ‘Thompson Seedless’ after 25 DAF. These expression data are similar to those derived by RT–PCR (Fig. 1a) and suggest that *VvMADS28* has a role in formation of the integument.

Gene function is determined not only by the spatio-temporal expression pattern, but also by the subcellular localization of the gene product. To examine the subcellular localization of *VvMADS28*, we expressed a green fluorescent protein (GFP)-tagged *VvMADS28* gene in arabidopsis protoplasts and found that the fluorescent protein product localized to the nucleus, as anticipated for a transcriptional regulator (Fig. 1c).

### Repression of *VvMADS28* in ‘Thompson Seedless’ ovules is associated with histone 3 Lys-27 trimethylation

**Figure 2:**
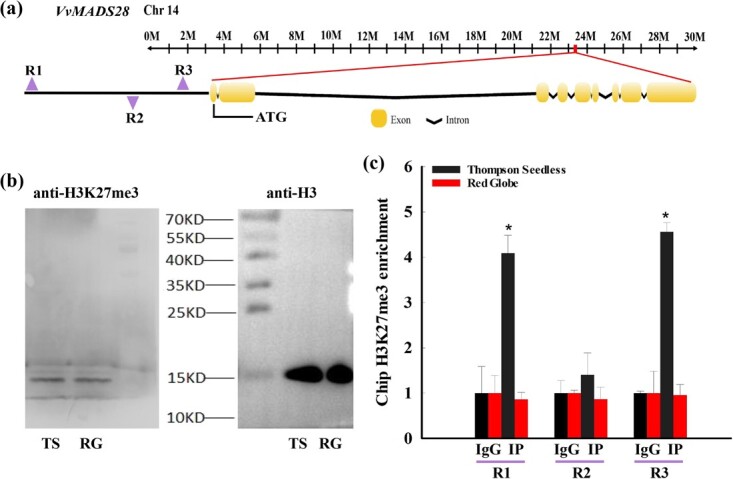
Analysis of H3K27me3 within the *VvMADS28* promoter region in ovules from ‘Thompson Seedless’ and ‘Red Globe’. **a** Diagram of the regions used for ChIP–qPCR assays. **b** Immunoblot analysis of overall levels of H3K27me3 and H3 in ‘Thompson Seedless’ and ‘Red Globe’. **c** H3K27me3 enrichment of the three *VvMADS28* promoter regions in ovules of ‘Thompson Seedless’ and ‘Red Globe’ as analyzed by ChIP–qPCR. ChIP values were normalized to their respective inputs. Error bars indicate ± standard deviation from three technical replicates. Statistical significance is denoted by an asterisk.

In multicellular organisms, histone 3 Lys-27 trimethylation (H3K27me3) serves a conserved role in transcriptional repression of key developmental genes. It has been reported that some ovule development genes in arabidopsis, such as *STK*, *INO*, *STM*, *LEC*, and *FUS3*, are modified by H3K27me3 [22, 23]. In ‘Pinot Noir’, H3K27me3 contributes to seed formation by inhibiting the biosynthesis of salicylic acid, thus precluding programmed cell death [24]. Our observation that *VvMADS28* was differentially expressed between the ovules of ‘Red Globe’ and ‘Thompson Seedless’ suggested that expression might be influenced by H3K27me3 within its promoter. Therefore, chromatin immunoprecipitation (ChIP)–qPCR assays were performed to assess H3K27me3 enrichment for three promoter regions: R1 (−2000/−1854), R2 (−866/−749), and R3 (−382/−249) (Fig. 2a). The results of Western blotting showed that the anti-H3K27me3 antibody and chromatin were of sufficiently high quality to be used for further study (Fig. 2b). RT–PCR revealed that both R1 and R3 showed a significant H3K27me3 enrichment in ‘Thompson Seedless’ compared with ‘Red Globe’ (Fig. 2c). This finding is consistent with the conserved role of H3K27me3 in gene repression, and with our observation that *VvMADS28* was relatively silenced in ‘Thompson Seedless’.

### Phenotypic observation of transgenic tomato plants overexpressing *VvMADS28*

**Figure 3:**
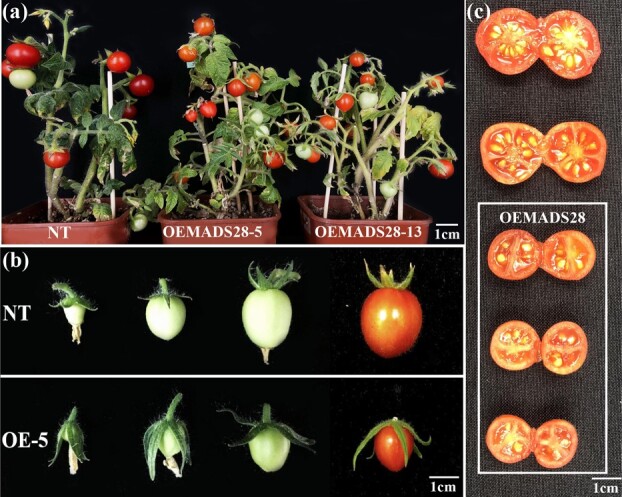
Functional analysis of transgenic tomato plants overexpressing *VvMADS28.***a** Phenotype of non-transgenic (NT) control plants and two *VvMADS28* overexpression lines. **b** Morphology of sepals and fruit from NT and *VvMADS28* overexpression line OE-5. **c** Cut fruit showing seed number for NT (top) and overexpression line OE MADS28. Scale bars are 1 cm in each panel.

We then expressed *VvMADS28* in transgenic tomato to evaluate the effects on fruit and seed development. VvMADS28*-*overexpression (OE-MADS28) plants did not show any obvious defects in growth, development, or physiology compared with the non-transgenic (NT) control plants during vegetative growth (Fig. 3a). However, floral sepals were greatly elongated, enclosing the floral buds at anthesis (Supplementary Data Fig. S1a–d) and elongating further during early fruit development (Supplementary Data Fig. S1e and f). Moreover, fruit of OE-MADS28 plants were smaller and contained fewer seeds (Fig. 3b and c). To quantify these phenotypes, average sepal length, fruit diameter, seed number, and seed length were determined from three independent OE-MADS28 lines (Table 1). Sepal length for the three OE-MADS28 lines was ~15.01 mm, while that for the NT was ~10.41 mm. The average fruit diameter for the OE-MADS28 lines was ~15.10 mm, whereas that for the NT was ~18.51 mm. Moreover, the average number of seeds per fruit for the OE-MADS28 lines ranged from 15 to 20, whereas that for NT was ~28. We did not observe any obvious difference in seed size between OE-MADS28 and NT plants.

**Table 1:** Statistical analysis of related traits in the *VvMADS28* overexpression lines.

Genotype	Sepal length	Fruit diameter	Average seeds	Seed length
	(mm)	(mm)	per fruit	(mm)
NT	10.41 ± 0.12	18.51 ± 0.15	28 ± 1.35	5.12 ± 0.23
OE28-5	15.01 ± 0.11	15.68 ± 0.32	20 ± 1.02	4.88 ± 0.09
OE28-13	14.38 ± 0.06	14.55 ± 0.09	18 ± 2.36	5.04 ± 0.18
OE28-15	15.65 ± 0.31	14.95 ± 0.27	15 ± 1.68	5.18 ± 0.13

Values are expressed as mean ± standard deviation. The number of samples used for each set of data was 5.

### Characterization of seed development in VvMADS28-RNAi fruit

**Figure 4:**
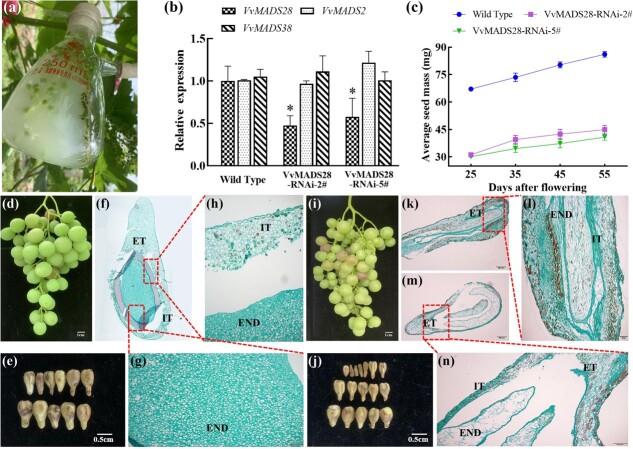
Transformation of grapevine and phenotype of VvMADS28-RNAi fruit and seed. **a***Agrobacterium*-mediated transient transformation. **b** Expression of *VvMADS28* and closely related genes in wild-type and two VvMADS28-RNAi lines. Error bars indicate ± standard deviation (*n* = 3). Statistical significance is denoted by an asterisk. **c** Average seed mass of wild-type and VvMADS28-RNAi lines. **d**, **e** Fruit and seed from wild-type. **f** Whole seed from wild-type. **g**, **h** Enlarged partial images of (**f**). **i**, **j** Fruit and seed from VvMADS28-RNAi line. **k**, **m** Seed from VvMADS28-RNAi plant. **l**, **n** Partial enlarged images of (**k**) and (**m**), respectively. END, endosperm; INT, integument; ET, endotesta.

Furthermore, we used *Agrobacterium*-mediated transient transformation to introduce a VvMADS28-RNAi construction into developing fruit of ‘Red Globe’ (Fig. 4a). qPCR showed that *VvMADS28* transcript accumulation was reduced in the ovules of the transgenic lines (VvMADS28-RNAi-2# and VvMADS28-RNAi-5#) compared with the NT, wild-type controls. To determine the specificity of the RNAi, we evaluated expression of *VvMADS38* and *VvMADS2,* which are the two genes in the grapevine genome most closely related to *VvMADS28*, in VvMADS28-RNAi lines, and found that expression of neither gene was impacted (Fig. 4b). These results indicated that the observed phenotype was due to loss of *VvMADS28* function. We found that, in VvMADS28-RNAi lines, the average seed mass was about half that of the wild-type (Fig. 4c), and the fruit and seeds from them were significantly reduced in size (Fig. 4d, e and 4i, j, respectively). As shown in Fig. 4f–h, the endosperm cells of the wild-type seeds were arranged in a compact and regular pattern, whereas those of VvMADS28-RNAi seeds were shriveled and irregularly arranged and failed to fill the embryo sac (Fig. 4k–n). The integument and endopleura cells of wild-type seeds were relatively thick and compact (Fig. 4h), but episperm cells of VvMADS28-RNAi lines were irregularly arranged and loosely packed (Fig. 4k–n). These results suggest that *VvMADS28* has a role in promoting normal seed formation.

### *VvERF98*, encoding an ethylene-response factor transcription factor, promotes *VvMADS28* expression in the seed

**Figure 5:**
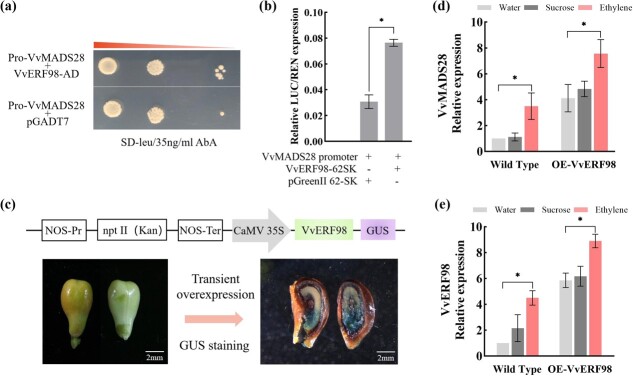
Analysis of upstream binding genes and activity of the *VvMADS28* promoter. **a** Analysis of the interaction between VvERF98 and the *VvMADS28* promoter using a Y1H system. **b** Relative LUC activity measured in *N. benthamiana* leaves using a dual-LUC reporter assay. Error bars indicate ± standard deviation (*n* = 6). Statistical significance is denoted by an asterisk. **c** Diagram of the *VvERF98* overexpression vector and transient overexpression in grape seeds. **d**, **e***VvMADS28* and *VvERF98* expression in the seeds of wild-type and *VvERF98*-overexpressing lines after treatment with water, sucrose, and ethylene.

To identify potential upstream regulators of *VvMADS28*, we carried out a yeast one-hybrid screen, using an ovule cDNA library in combination with an ~2 kb *VvMADS28* promoter region. This resulted in the identification of multiple cDNA clones corresponding to *VvERF98* (gene ID 100241203), encoding an ethylene-response factor (ERF). *VvERF98* was expressed in the two cultivars during ovule development in a pattern similar to that of *VvMADS28* (Supplementary Data Fig. S2a). As shown in Fig. 5a, when a plasmid expressing *VvERF98* was transformed into the yeast one-hybrid (Y1H) strain, cells harboring the *VvMADS28* promoters grew well with aureobasidin A, whereas cells co-transformed with the empty pGADT7 vector did not. These results suggested that *VvERF98* binds to the promoter of *VvMADS28*. Moreover, a dual-luciferase (LUC) reporter assay in *Nicotiana benthamiana* leaves showed that the relative intensity of LUC was markedly increased with co-transformation of pVvMADS28-LUC with 62SK-VvERF98, compared with that of the control transformed with pVvMADS28-LUC alone (Fig. 5b). To gain additional support, a VvERF98-GUS overexpression vector was introduced into grapevine seeds (Fig. 5c). GUS staining indicated that *Agrobacterium* solution had penetrated into the grape seeds. Expression of *VvMADS28* was markedly increased in the seeds of VvERF98-GUS plants (Fig. 5d). These results showed that *VvMADS28* can be positively regulated by *VvERF98.*

The identification of *VvERF98* as an ERF suggests that *VvMADS28* expression may be responsive to ethylene signaling. To test this, we examined the transcriptional response of *VvERF98* and *VvMADS28* to ethephon. As can be seen in Fig. 5d and e, both *VvERF98* and *VvMADS28* were more highly upregulated in the seed after treatment with 10 mM ethephon compared with control plants treated with water or sucrose alone. Collectively, these results indicate that *VvERF98* acts as an upstream transcriptional regulator of *VvMADS28* in seed development and is responsive to ethylene signaling.

### DNA affinity purification sequencing analysis

**Figure 6:**
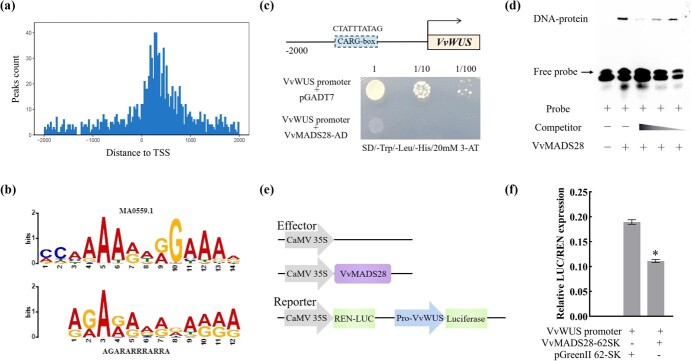
DAP-seq analysis and potential targets of VvMADS28 in grapevine. **a** Distribution of *VvMADS28* binding sites in 2-kb flanking sequences around genic peak summits. **b** Significantly enriched motif sequence of *VvMADS28* binding sites specifically detected by DAP-seq. **c** Analysis of the interaction between VvMADS28 and the *VvWUS* promoter using a Y1H system. **d** EMSA of interaction between recombinant VvMADS28 protein and the CArG region of the *VvWUS* promoter. **e** Dual-reporter and effector plasmids utilized in the dual-LUC reporter test. **f** Relative LUC/REN expression in *N. benthamiana* leaves. Co-expression of *VvMADS28* decreased the expression of the LUC reporter gene driven by the *VvWUS* promoter. Data are from three biological replicates, with error bars indicating the standard deviation. Statistical significance is denoted by an asterisk.

To identify potential targets of *VvMADS28* in the grapevine genome, we performed a DNA affinity purification sequencing (DAP-seq) assay, using a tagged version of *VvMADS28* as bait. The results from two biological replicates showed that the average unique mapped ratio of reads produced by VvMADS28-bound DNA in the grapevine reference genome was 41.51%, and the average mapped ratio of reads was 90.40% (Supplementary Data Table S2). Moreover, as shown in Supplementary Data Fig. S3a and b, *VvMADS28* binding peaks were distributed across all chromosomes. We identified 760 intergenic peaks (located in the region beyond 2 kb upstream of the gene initiation transcription site) as well as 726 promoter peaks (located within 2 kb upstream of the gene transcription start site). A total of 238 peaks were identified in both replicates and were designated as high-confidence *VvMADS28* binding sites. These were mainly located near transcription start sites (Fig. 6a), and motif enrichment analysis identified the CArG sequence CC(A/T)_6/8_GG as significantly enriched (Fig. 6b). KEGG (Kyoto Encyclopedia of Genes and Genomes) enrichment analysis of potential *VvMADS28* target genes revealed over-representation for biological metabolism, the biosynthesis pathway, and the MAPK signaling pathway (Supplementary Data Fig. S3c).

### Potential targets of *VvMADS28* in grapevine

Several of the high-confidence *VvMADS28* binding sites were proximal to genes homologous to ovule development genes identified in arabidopsis, including *WUSCHEL-related homeobox 1* (*WUS*, VIT_217s0000g02460), *HAIKU* (*IKU*, VIT_202s0012g01280), *DA* (VIT_213s0019g02430), and *INNER NO OUTER* (*INO*, VIT_201s0127g00330). The promoter sequences of all these four genes contained at least one CArG [CC(A/T)_6/8_GG] site. To assess potential interaction between VvMADS28 protein and DNA sequences within these four promoters, we carried out Y1H experiments. We found that VvMADS28 interacted only with the promoter of the grapevine *WUS* homologue *VvWUS* (Fig. 6c). To further confirm that VvMADS28 can specifically bind to CArG (CTATTTATAG) site(s), we carried out an electrophoretic mobility shift assay (EMSA). As shown in Fig. 6d, recombinant His-tagged VvMADS28 showed specific interaction with the biotin-labeled fragment of the *VvWUS* promoter. Furthermore, dual-LUC assays in tobacco leaves showed that the potential interaction of VvMADS28 with the promoter of *VvWUS* led to a nearly 1.5-fold decrease in the LUC/REN ratio (Fig. 6e and f). These results suggest that *VvMADS28* inhibited the expression of grapevine *VvWUS* by binding to the CArG (CTATTTATAG) site in the *VvWUS* promoter.

### VvMADS28 physically interacts with VvMADS5

**Figure 7:**
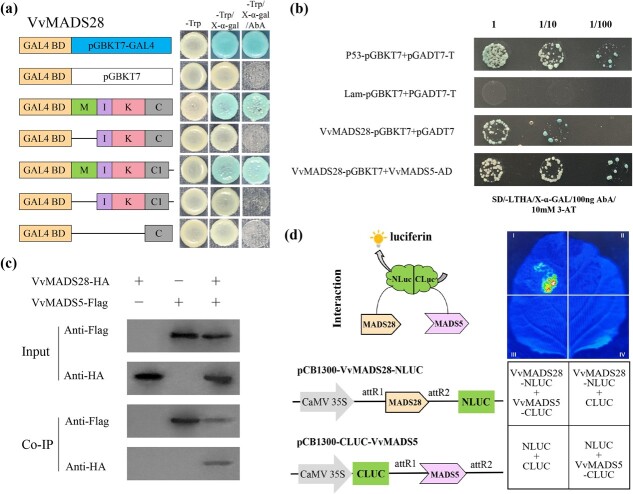
Interaction between VvMADS28 and VvMADS5. **a** Activation potential of VvMADS28 and respective deletions in yeast. **b**–**d** Y2H analysis (**b**), co-immunoprecipitation (**c**), and split-LUC (**d**) assays were performed to evaluate the interaction between VvMADS28 and VvMADS5.

Given the developmental complexity of the ovule, we anticipated that VvMADS28 cooperates with numerous additional transcription factors. We used a yeast two-hybrid (Y2H) approach to discover proteins that physically interact with VvMADS28. Because expression of full-length *VvMADS28* containing the MIKC region was independently able to activate the reporter gene, we engineered a non-activating *VvMADS28* sequence by successive deletions of its carboxyl-terminal sequence. The longest *VvMADS28* sequence lacking activation potential was used as the bait. Screening of a grapevine ovule cDNA library resulted in the identification of VvMADS5 (VIT_00010218001). Further Y2H experiments in yeast confirmed interaction of VvMADS28 and VvMADS5 (Fig. 7b). To gain further support, we used a co-immunoprecipitation (Co-IP) assay, with a VvMADS28-HA fusion protein in combination with a VvMADS5-Flag recombinant protein, transiently co-expressed in *N. benthamiana*. The result showed that VvMADS28 and VvMADS5 were efficiently co-immunoprecipitated (Fig. 7c). Furthermore, a split-LUC complementation assay showed that co-expression of VvMADS28-NLUC and VvMADS5-CLUC in *N. benthamiana* leaves generated a clear signal of interaction (Fig. 7d).

### Functional characterization of *VvERF98*, *VvMADS5*, and *VvWUS* in arabidopsis

**Figure 8:**
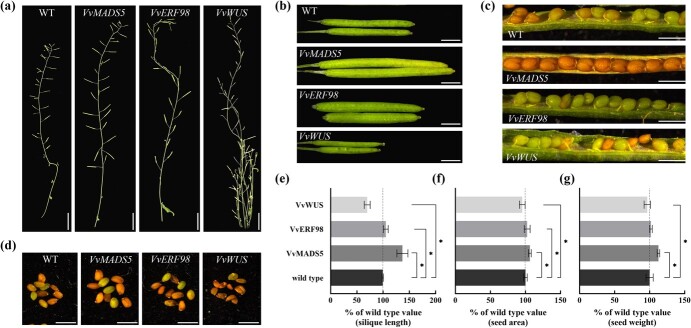
Phenotypic analysis of non-transgenic wild-type (WT) arabidopsis and transgenic arabidopsis constitutively expressing *VvERF98*, *VvMADS5*, and *VvWUS*. **a** Inflorescence stalks. **b** Siliques. **c** Opened siliques. **d** Seeds. Scale bars are 1 cm in (**a**), 2 mm in (**b**), and 1 mm in (**c**) and (**d**). **e**–**g** Quantification of silique length (**e**), projective area (**f**), and seed weight (**g**). Error bars in (**e**–**g**) indicate means ± standard deviation relative to the respective WT values, set as 100%. *P < .05 (one-way ANOVA).

To obtain additional evidence to support the biological relevance of the interactions among *VvERF98*, *VvMADS5*, and *VvWUS* for seed development, we conducted preliminary functional analyses of these three genes in arabidopsis. For each gene, stable transgenic lines were engineered constitutively expressing the coding sequence under control of the 35S CaMV promoter. In comparison with non-transgenic plants, OE-VvMADS5 plants had larger leaves and siliques, and seeds showed increased size and weight (Fig. 8 and Supplementary Data Fig. S4). These results indicate that *VvMADS5* plays an important role in ovule and organ development. Similarly, seeds of OE-VvERF98 plants were larger than those of control plants (Fig. 8). This suggests that *VvERF98* is involved in ovule development. In contrast, OE-VvWUS plants produced twisted leaves and tufted branches, and the siliques and seeds were smaller than those of the controls (Fig. 8 and Supplementary Data Fig. S4). In addition, it is worth mentioning that gene expression patterns provide clues to this functional differentiation; *VvWUS* mRNA was relative highly accumulated in the ovule of ‘Thompson Seedless’; its expression pattern was the opposite of *VvMADS5* and *VvERF98* (Supplementary Data Fig. S2a–c). These results also suggest that the function of *VvWUS* may be different from that of *VvMADS5* and *VvERF98.*

## Discussion

Duplication of MADS-box genes is a common phenomenon during plant evolution, and not only gives rise to functionally redundant genes, but also permits changes in expression pattern or protein function, leading to diversification of developmental processes [25, 26]. Phylogenetic analysis shows that *VvMADS28* belongs to the *AP1*/*FUL* subfamily (Supplementary Data Fig. S5), and therefore is a class A gene according to the ABC model. The general function of *AP1* in arabidopsis is to help direct formation of sepals and petals. Interestingly, we found that *VvMADS28* was highly expressed in flowers, sepals, petals, and fruit, analogous to the expression pattern of its arabidopsis counterparts. In addition, the tomato genome contains a *VvMADS28* homolog, designated *FUL1* (also known as *TDR4*), which is primarily involved in fruit ripening and shares a similar expression pattern to *VvMADS28*, indicating that homologous genes from different species may perform a conserved function.

We found that *VvMADS28* was relatively highly expressed in floral organs as well as in the ovule during later development stages of ‘Red Globe’, indicating that *VvMADS28* not only is involved in flower morphogenesis but also participates in development of the integument. ChIP–qPCR assays indicated that the enrichment of the H3K27me3 repressive marker in the promoter region of *VvMADS28* was much higher in ‘Thompson Seedless’ than in ‘Red Globe’, potentially explaining the differential expression of *VvMADS28* in the ovules of these two cultivars. On the other hand, the bigger seeds produced by overexpression of *VvERF98* (Fig. 8) as well as the differential expression pattern of *VvERF98* in ‘Thompson Seedless’ and ‘Red Globe’ (Supplementary Data Fig. S2a) indicate that *VvERF98* plays a guiding role in the expression and phenotypic regulation of *VvMADS28*. However, this inference warrants further verification in stable transgenic grapevine lines.

When ectopically expressed in tomato to high levels, *VvMADS28* promoted sepal growth, but repressed fruit growth. Likewise, overexpression of the homologous *SlFUL* in tomato led to smaller fruit, due to reduced division and expansion of pericarp cells, suggesting that *VvMADS28* may affect fruit development [27]. However, no obvious effect of *SlFUL* overexpression on sepal morphology was noted. In our study, seed size was not greatly affected by ectopic expression of *VvMADS28*, but seed numbers were reduced. This could result from limitation of maternal resources. On the other hand, our results showed that inhibition of episperm and endosperm cell development correlated with reduced nuclear proliferation and reduced seed size in VvMADS28-RNAi transgenic lines. This is consistent with the reduction in seed mass observed in arabidopsis *ful* mutants [20]. Interestingly, the expression of two other genes from *AP1*/*FUL* group, *VvMADS38* and *VvMADS2*, was not impacted in VvMADS28-RNAi lines. Moreover, the expression patterns of *VvMADS38* and *VvMADS2* during ovule development were obviously different from *VvMADS28* (Supplementary Data Fig. S2d and e), suggesting that the different *AP1*/*FUL* genes have evolved by acquiring temporal-specific expression patterns and may perform different functions.

The MADS-box superfamily can be divided into Type I and Type II sequences [28]. Type II genes, including *AP1*, *FUL*, and *VvMADS28*, have been investigated extensively; however, there is more limited information about potential function(s) of Type I genes [29]. In grapevine, previous studies indicated that Type I genes are widely expressed in roots, stems, leaves, flowers, tendrils, fruits, and ovules [30], suggesting general and important functions. In general, Type II proteins interact with other Type II proteins, while interactions between Type I and II proteins are rarely reported [31]. We found that VvMADS28 can interact with a Type I protein of the Mβ clade, VvMADS5. VvMADS5 is closely related to the arabidopsis Type I MADS domain protein AGL62. AGL62 was shown to be a key regulator of endosperm cellularization, which correlates with the extent of nuclear proliferation and may influence seed size [32]. In this study, heterologous overexpression of *VvMADS5* in arabidopsis produced larger seeds and organs (Fig. 8 and Supplementary Data Fig. S4b and c). Therefore, we can reasonably anticipate it has a role in ovule development analogous to that of *AGL62*. Correspondingly, the expression of *VvMADS5* was also significantly reduced in VvMADS28-RNAi lines, associated with abnormal ovule development (Supplementary Data Fig. S2g). This was associated with reduced nuclear proliferation, similar to the phenotype conferred by loss of function of *AGL62* in arabidopsis. Decreased expression of *VvMADS5* led to precocious cellularization, and in VvMADS28-RNAi transgenic lines development of the embryo and embryo sac was inhibited, resulting in the collapse of the endosperm and fewer and smaller seeds. These results suggest that *VvMADS5* and *VvMADS28* are important for maintaining normal ovule development.

DAP-seq analysis can provide a simple access to understanding transcriptional regulators. MADS-box proteins have been found to generate a relatively small DAP-seq dataset, compared with bZIP or NAC proteins [33]. Our DAP-seq analysis of *VvMADS28* generated specific peaks and binding-site motifs (CArG) consistent with previous reports [34], indicating that our results are reliable. Meanwhile, four candidate genes related to ovule development were selected from the DAP-seq results. *HAIKU* (*IKU*) is a key gene regulating endosperm development in arabidopsis. It has been reported that *iku1*/*iku2*/*miniseed3* triple mutants show precocious endosperm cellularization that leads to premature developmental arrest. The HAIKU pathway regulates seed size in collaboration with auxin and cytokinin [3]; *DA1* is a receptor gene that affects integument/seed coat development by controlling cell proliferation. The arabidopsis *da1-1* line produced larger organs, indicating that *DA1* inhibited the development of seeds [35]; *INO* encodes a YABBY transcription factor; *ino* mutants show developmental defects in the integument, and this phenotype can be rescued by transgenic expression of *VvINO* [36]; *WUS* encodes a homeodomain protein, and was originally identified as a central regulatory gene in shoot and flower meristem development [37]*.* Moreover, *WUS* activity is required to initiate the transformation from chalaza to integument and is involved in the regulation of ovule development [38]. We performed Y1H assays to evaluate binding of VvMADS28 with promoter regions from each of these four genes, and only the interaction with *VvWUS* was identified (Supplementary Data Fig. S6). It has been noted that some DNA-binding proteins require other co-factors or interacting proteins for binding to their *cis*-element targets [39, 40]. That we did not observe Y1H interaction of VvMADS28 with the remaining three genes identified by DAP-seq may be due to the requirement for specific transcriptional co-regulators not found in yeast cells, or plant-specific post-translational modification of VvMADS28.

Furthermore, we analyzed the expression pattern of *VvWUS* during seed development. *VvWUS* mRNA was relatively highly expressed in the ovules of ‘Thompson Seedless’, and in an opposite pattern to that of *VvMADS28* (Supplementary Data Fig. S2c). Interestingly, expression of *VvWUS* was increased in VvMADS28-RNAi seeds. In the seeds of plants overexpressing *VvERF98*, *VvMADS28* was obviously upregulated; however, *VvWUS* mRNA remained constant (Supplementary Data Fig. S2f). We suspect that *VvWUS* activity is dependent on *VvMADS28* expression, and upregulation of *VvWUS* was only observed when the *VvMADS28* gene was inactive. A previous report suggests that MADS activities balance ovule identity activity by regulating WUS [41]*.* Therefore, the presence of WUS signaling in ovules might reflect a short-range conserved signaling module, which is employed in ovules to coordinate the development of neighboring cell groups [38]. In addition, we found that overexpression of *VvWUS* in arabidopsis resulted in aberrant development, including distorted leaves and smaller seeds (Supplementary Data Fig. S4c and Fig. 8). Similarly, it has been reported that overexpression of *WUS* in tobacco causes severe leaf curl and reduced seed germination. These results suggest that strict regulation of *WUS* expression may play an important role in maintaining normal development. In view of the gene expression pattern and the negative regulatory relationship between *VvMADS28* and *VvWUS*, we hypothesize that *VvMADS28* expression in the ovule of ‘Red Globe’ limits *VvWUS* expression to maintain normal ovule development*.* The low expression of *VvMADS28* in ‘Thompson Seedless’ associated with histone methylation may result in derepression of *VvWUS* and inhibition of seed development. Although this hypothesis is grounded in our observation of the molecular regulatory relationship between *VvMADS28* and *VvWUS*, it still needs further testing. MADS-box and WUS transcriptional regulators are anticipated to have many additional roles in the developing ovule, and regulation of these processes is an area of active investigation.

**Figure 9:**
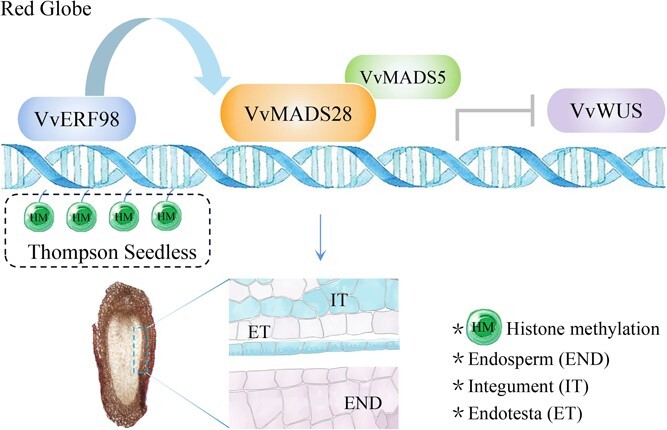
A model for the regulatory network of seed development involving *VvMADS28*. *VvMADS28* is positively regulated by *VvERF98*. The relatively low expression level of *VvMADS28* in ‘Thompson Seedless’ is associated with increased H3K27me3 repressive marks within its promoter region. VvMADS28 protein specifically binds to the *VvWUS* promoter and VvMADS28 interacts with VvMADS5 to repress *VvWUS*. The maintenance of *VvMADS28* and *VvWUS* expression homeostasis may affect seed development.

In conclusion, we put forward the model shown in Fig. 9. The differential enrichment of histone methylation is associated with the differential expression of *VvMADS28* in the ovules of these two cultivars. H3K27me3 within the *VvMADS28* promoter in ‘Thompson Seedless’ inhibits the expression of *VvMADS28.* In addition, the upstream transcription factor *VvERF98* promotes the expression of *VvMADS28.* Meanwhile, VvMADS28 interacts with the Type I MADS protein VvMADS5, and VvMADS28 protein specifically binds to the *VvWUS* promoter. The maintenance of VvMADS28-VvMADS5 dimer and *VvWUS* expression homeostasis may affect seed development.

## Materials and methods

### Plant material

Plant structures were dissected from one seeded (‘Red Globe’) and one seedless (‘Thompson Seedless’) cultivar maintained in the Grape Germplasm Resources of Northwest A&F University, Yangling, China (34°20′N, 108°24′E). ‘Micro-Tom’, a control tomato, and transgenic tomato were grown in a containment greenhouse at temperatures between 23 and 25°C [42].

### Transient suppression of *VvMADS28* in grape berries and heterologous overexpression of *VvMADS28*, *VvMADS5*, *VvERF98*, and *VvWUS* in model plant

The plasmid pHellsgate2 was utilized to construct the RNA silencing vector for *VvMADS28* based on gateway recombination technology (Invitrogen). When grapevine plants were flowering, infiltration was performed by submerging an inflorescence into the *Agrobacterium* suspension and applying a vacuum for 10 minutes. The same plants were subjected to transformation once a week for 4 weeks.

The overexpression vector pCAMBIA2300-flag-VvMADS28 was created and introduced into *Agrobacterium tumefaciens* strain GV3101 as elaborated previously [13]. Tomato (‘Micro-Tom’) plants were subjected to transformation by the cotyledon disk method [11, 43].

For heterologous expression in transgenic arabidopsis, VvMADS5/VvERF98/VvWUS coding sequences were engineered into the pCAMBIA2300-GFP vector. Transformation of arabidopsis used the floral dip method [44]. Seeds were collected after the transformed plants matured and screened on nutrient agar medium containing 50 mg/l kanamycin. *T*_3_ plants were used for characterization.

### Morphological observation and measurements of arabidopsis seeds

The images of siliques and seeds were obtained using a stereo microscope. Seed area was determined with ImageJ software according to the method described earlier [45]. Seed weight was measured with 600 seeds using a stereo microscope (Leica MZ10F).

### Real-time quantitative polymerase chain reaction

Plant RNA extraction was performed as described previously [13]. First-strand cDNA was synthesized using the Evo M-MLV RT Reagent Kit (Accurate Biotechnology Co., Ltd, Hunan, China). Quantitative RT–PCR was conducted on a StepOne™ instrument in 20-μl reaction volumes. Grapevine *EF1-α* (GenBank accession number EC931777) and *ACTIN* (EC969944) served as internal standards. The 2^-ΔΔCT^ method was used to determine expression levels using data from three biological replications [46]. Sequences of oligonucleotide primers can be found in Supplementary Data Table S1.

### *In situ* hybridization

Ovules were preserved and embedded in paraffin at various stages of development. Sections (6–8 μm) were cut using a microtome (Leica, Wetzlar, Germany). Digoxigenin (DIG)-labeled probes were synthesized by *in vitro* transcription from *VvMADS28* cDNA using sequence-specific primers and T7 or SP6 RNA polymerase, with a DIG RNA Labeling Kit (Roche, Basel, Switzerland). The sections were subjected to hybridization as described elsewhere [24, 47]. Hybridization signals were detected by colorimetry with an anti-DIG-AP antibody. Sections were photographed under a light microscope (Nikon Eclipse Ci).

### Subcellular localization of *VvMADS28*

*VvMADS28* cDNA was inserted into the pHBT-GFP-NOS green fluorescence expression vector to create VvMADS28-pHBT-GFP-NOS. The recombinant expression plasmid was introduced into arabidopsis protoplasts using a previously described method [48, 49]. Green fluorescence was scanned using a confocal laser scanning microscope (Leica TCS-SP8 SR). The nucleus was marked with m-Cherry red fluorescent protein.

### Yeast two-hybrid assay

The *VvMADS28* cDNA was inserted into the pGBKT7 plasmid to yield VvMADS28-BD, which was introduced into the yeast strain Y2H Gold. A cDNA library was prepared from ‘Thompson Seedless’ ovules at various developmental stages as an outservice (TaKaRa Bio). The cDNA library was mated with Y2H-VvMADS28-BD as described in the Matchmaker user manual, and colonies were selected on synthetic dropout medium. To directly evaluate protein–protein interactions, VvMADS5-AD and VvMADS28-BD were then co-transformed into Y2H Gold and colonies were screened on dropout medium. A more detailed description can be found in the Matchmaker™ Gold Yeast Two-Hybrid System (TaKaRa Bio) user manual.

### Yeast one-hybrid assay

The pAbAi system was used to verify the interaction between *VvERF98* and the promoter of *VvMADS28*, using the protocols specified in the Matchmaker™ Gold Yeast One-Hybrid Library Screening System (Clontech) user manual. The pHIS2 system was used to verify the interaction between *VvMADS28* and the promoter of *VvWUS*. Briefly, a promoter segment of *VvWUS* was inserted into the pHIS2 reporter vector. *VvMADS28* was inserted into the pGADT7 vector. The recombinant plasmids were then co-transformed into Y187. The non-modified pGADT7 plasmid was used as a negative control. Other steps were carried out as specified by the manufacturer (TaKaRa Bio).

### Split luciferase assay

The *VvMADS5* coding sequence was cloned into the pCB1300-CLUC vector to create pCB1300-CLUC-VvMADS5. The full-length *VvMADS28* coding sequence without the stop codon was recombined into pCB1300-NLUC to generate pCB1300-NLUC-VvMADS28. The resulting plasmids were introduced into *A. tumefaciens* strain GV3101, and the mixed bacterial solution was transiently transformed into *N. benthamiana* leaves. At 48 hours after infiltration, LUC activity was detected using a charge-coupled device (CCD) camera [49].

### Dual-luciferase assay

Approximately 2-kb promoter segments of *VvMADS28* and *VvWUS* were recombined into the transient gene expression vector pGreenII 0800-LUC. *VvERF98* and *VvMADS28* were inserted into pGreenII-62SK under control of the CaMV 35S promoter. The plasmids were transformed into *Agrobacterium* GV3101 pSoup cells and infiltrated into *N. benthamiana* leaves. After 48 hours, REN and LUC activities were quantified using the Dual-Luciferase Reporter Assay System Kit (Beyotime, China). Six measurements were conducted per assay. DNA–protein interaction was quantified by calculating the LUC/REN ratio.

### Co-immunoprecipitation assays

VvMADS28-HA and VvMADS5-Flag were created by cloning the *VvMADS28* and *VvMADS5* coding sequences without stop codons into the pEarleyGate202-Flag and pEarleyGate201-HA vectors, respectively. Total proteins were then obtained from the infiltrated *N. benthamiana* leaves using a previously reported method [50]. Extracts were incubated with 4 μl of Flag antibody for 2 hours at 4°C. After addition of G-Sepharose, the samples were incubated for an additional 12 hours. Beads were then washed three times for 10 minutes each time. Protein samples were resolved by SDS–PAGE and transferred to polyvinylidene fluoride (PVDF) membranes (0.45 μm). After transfer, membranes were inoculated with specific antibodies (anti-HA and anti-Flag), and membranes were subjected to luminescence imaging (Alliance Q9 Advanced).

### Chromatin immunoprecipitation

ChIP experiments were conducted following an improvised version of a previously reported protocol [51, 52]. Immunoprecipitation of chromatin from ‘Red Globe’ or ‘Thompson Seedless’ ovules utilized an anti-H3K27me3 mouse monoclonal antibody (1:1000, Abcam ab6002), anti-H3 antibody (positive control), and anti-IgG. Three aliquots for incubation without antibodies were used as input. After purification and recovery, immunoprecipitated DNAs were used as templates. Gene-specific primers are listed in Supplementary Data Table S1. Three technical measurements were conducted per sample. CT values obtained from DNA immunoprecipitated using IgG only as template were used to determine the level and significance of H3K27me3 enrichment.

### Electrophoretic mobility shift assay

For production of recombinant, His-tagged VvMADS28 protein, the *VvMADS28* cDNA was engineered into the pET-32a vector, introduced into *Escherichia coli* BL21 cells, and induced at 16°C for 12 hours. Protein was recovered and purified using a His-Tagged protein purification kit. Double-stranded, 5′-end biotin-labeled oligonucleotides containing 3× tandem repeats of the putative binding sites were created by a commercial service. Identical, non-labeled oligonucleotides were employed as competitor probes. The LightShift Chemiluminescent EMSA Kit (ThermoFisher) was used for the EMSA according to the manufacturer’s instructions.

### DNA affinity purification sequencing analysis

DAP-seq was performed as an outservice by Bluescape Scientific, Hebei, China. Genomic DNA (gDNA) was extracted from leaves, and a DAP library was constructed after fragmentation of the gDNA. Recombinant VvMADS28 protein was obtained by engineering the *VvMADS28* cDNA into the pGEX 4 T expression vector, followed by expression and purification following the manufacturer’s specifications (Pierce™ Glutathione Magnetic Agarose Beads, Thermo Fisher). VvMADS28 protein and the gDNA library were incubated *in vitro* and DNA bound to VvMADS28 was isolated as described elsewhere [53, 54]. DNA obtained after affinity purification and elution was subjected to paired-end sequencing on an Illumina HiSeq platform. Quality-filtered reads were aligned to a Grapevine genome sequence (https://urgi.versailles.inra.fr/Species/Vitis/Annotations) by Bowtie2 [55]. Conserved motifs within peak regions were identified using MEME [56].

## Supplementary Material

Web_Material_uhad070

## Date availability

All relevant data are included in the article and its supporting materials.
