# Supplementary material for: Control of ovule development in Vitis vinifera by VvMADS28 and interacting genes
Source: Hortic Res. 2023 Apr 13;10(6):uhad070. doi: 10.1093/hr/uhad070 (PMC10244803; doi:10.1093/hr/uhad070)
Supplement: Web_Material_uhad070 [file web_material_uhad070.docx]

Table. S1 Oligonucleotide primers used in this study

| Gene | Vector |  | Primer(5'-3') | Purpose |
| --- | --- | --- | --- | --- |
| *VvActin* |  | F | GATTCTGGTGATGGTGTGAGT | Gene expression |
|  |  | R | GACAATTTCCCGTTCAGCAGT |  |
| *VvEF1-α* |  | F | AGGAGGCAGCCAACTTCACC |  |
|  |  | R | CAAACCCTGCATCACCATTC |  |
| *VvMADS28* |  | F | TTCTGTGCTGTGCGATGC |  |
|  |  | R | CCTTCTTTGCTAATGCGTTG |  |
| *VvMADS5* |  | F | GAAAGAGTAAGGGTCGCCA |  |
|  |  | R | TACCAGCATTTGTAAGAGG |  |
| *Chip-F1* |  | F | CCTCAGGAGCAGAGAACCC | Chip-qPCR |
|  |  | R | AAACATCTGTTCTTACCCCACT |  |
| *Chip-F2* |  | F | TCACCCAGAAGATTTCAGGA |  |
|  |  | R | TCTTTTCATGTCCATTGCTTG |  |
| *Chip-F3* |  | F | CCCAAAACGAGACAGTAACG |  |
|  |  | R | ATATAATTGGGAAGGGGGG |  |
| *VvMADS28* | Digoxin marker | F | DIG-ACCACTCCTCTCTCTCTGCTT | In situ hybridization |
|  |  | R | DIG-ACCACAAAATTGCAGAAACCCT |  |
| *VvMADS28* | pCAMBIA2300-flag | F | CGAGCTC ATGGGGAGAGGAAGGGTTCAGTTGA | Genetic  transformation |
|  |  | R | GCTCTAGA TTCATTGACGTGGCGCAGCATCCAT |  |
| *VvMADS28* | pEarleyGate201-YN-HA | F | ACAAGTTTGTACAAAAAAATGGGGAGAGGAAGGGTTC | CoIP |
|  |  | R | CACCACTTTGTACAAGAATTCATTGACGTGGCGCAG |  |
| *VvMADS5* | pEarleyGate202-YC-Flag | F | ACAAGTTTGTACAAAAAAATGGCTACTATAAGAAAGAGTAAGG |  |
|  |  | R | CACCACTTTGTACAAGAAATTGGTGTTGGTAGTATTTGAGG |  |
| *Pro-VvWUS* | pAbAi | F | ACGACTCACTATAGGGCGAATTCTTGAACTCATATTTGCCGG | Yeast one hybrid |
|  |  | R | TCGATTCGCGAACGCGTGAGCTCTGTAAGTTGCTTTGCTTTGA |  |
| *VvERF98* | pGADT-7 | F | CGGAATTCCG ATGGAAGAACACCCTAAAGGGA |  |
|  |  | R | CGGGATCCCG TTACTTCTCTGAAGTCTCAAGAAGG |  |
| *VvMADS28* | pGADT-7 | F | GCCATGGAGGCCAGT GAATTC ATGGGGAGAGGAAGGGTTCAGTTGA |  |
|  |  | R | CAGCTCGAGCTCGAT GGATCCTTATTCATTGACGTGGCGCAGCATC |  |
| *Pro-*  *VvMADS28* | pGreenII-0800 | F | CTATAGGGCGAATTGGGTACC AATGATAGACCTTGTATCCCCCAA | Dual-luciferase |
|  |  | R | TGTTTTTGGCGTCTTCCATGG TTGCGCTCCTTTCTTTTCTCCC |  |
| *VvERF98* | pGreenII-62SK | F | CGGGATCCCG ATGGAAGAACACCCTAAAGGGA |  |
|  |  | R | CGGAATTCCG TTACTTCTCTGAAGTCTCAAGAAGG |  |
| *Pro-VvWUS* | pGreenII-0800 | F | CTATAGGGCGAATTGGGTACC TTGAACTCATATTTGCCGGAAAATT |  |
|  |  | R | TGTTTTTGGCGTCTTCCATGG TGTAAGTTGCTTTGCTTTGAAGGTG |  |
| *VvMADS28* | pGreenII-62SK | F | CGCTCTAGAACTAGTGGATCCATGGGGAGAGGAAGGGTTCAGTT |  |
|  |  | R | GATAAGCTTGATATC GAATTCTTATTCATTGACGTGGCGCAGCATC |  |
| *VvMADS28* | RNAi | F1 | CGGAATTC TCGGAGCTTCAGAAGAAGGA | Transient transformation |
|  |  | F2 | CCATCGAT TCCGTCTTCCTCTACAGCTC |  |
|  |  | R1 | GGGGTACC TCCGTCTTCCTCTACAGCTC |  |
|  |  | R2 | GCTCTAGA TCGGAGCTTCAGAAGAAGGA |  |

Table. S2 Statistics of DAP-seq alignment

| Sample | Unique mapped ratio | Mapped ratio |
| --- | --- | --- |
| Input | 41.27% | 90.90% |
| VvMADS28-1 | 41.30% | 90.35% |
| VvMADS28-2 | 41.73% | 90.44% |


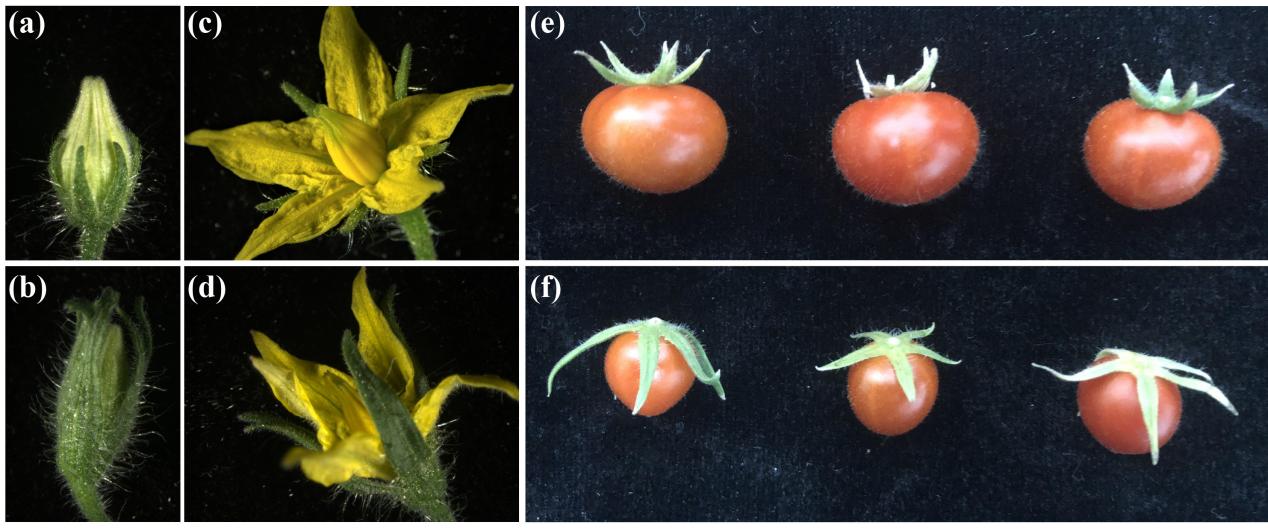


Figure. S1 Phenotypic comparison of petals, sepals and fruits between VvMADS28-overexpression and no-transgenic lines.

Phenotypic observation of flower bud (a), petal/sepals (c) and fruits (e) from no transgenic line. Phenotypic observation of flower bud (b), petal/sepals (d) and fruits (f) from VvMADS28-overexpression line.


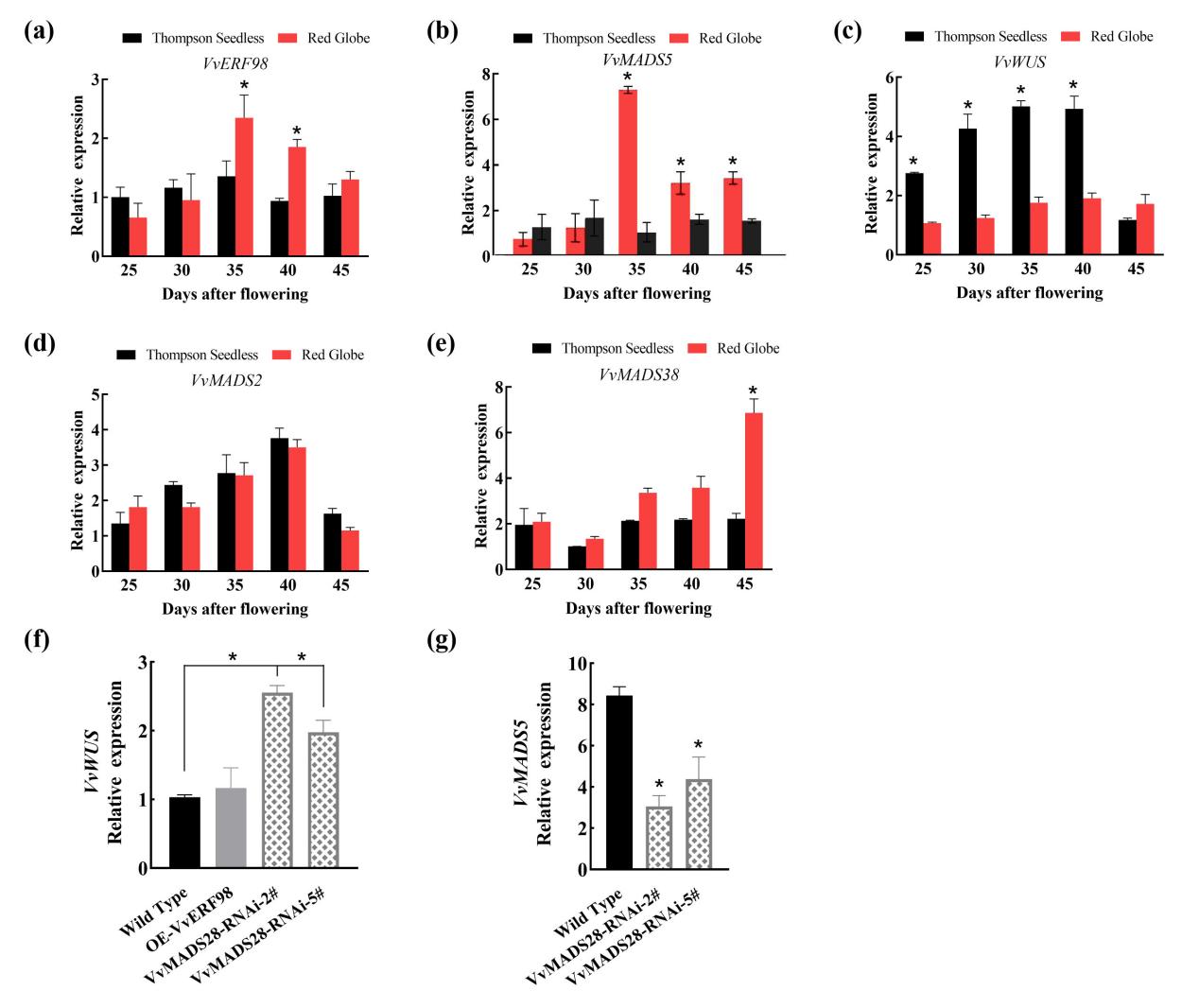


Figure. S2 Quantitative expression of related genes.

(a-c) Expression patterns of *VvERF98*, *VvMADS5*, and *VvWUS* in ovule at progressive developmental stages in 'Red Globe' and 'Thompson Seedless'. (d-e) Expression patterns of *VvMADS28* closely related genes in ovule at progressive developmental stages in 'Red Globe' and 'Thompson Seedless' . (f) Effect of VvMADS28-RNAi suppression plant on *VvWUS*. (g) Effect of VvMADS28-RNAi suppression plant on *VvMADS5*. Values are means ± SD of three biological replicates; * represent significance at P < 0.05 based on one-way ANOVA.


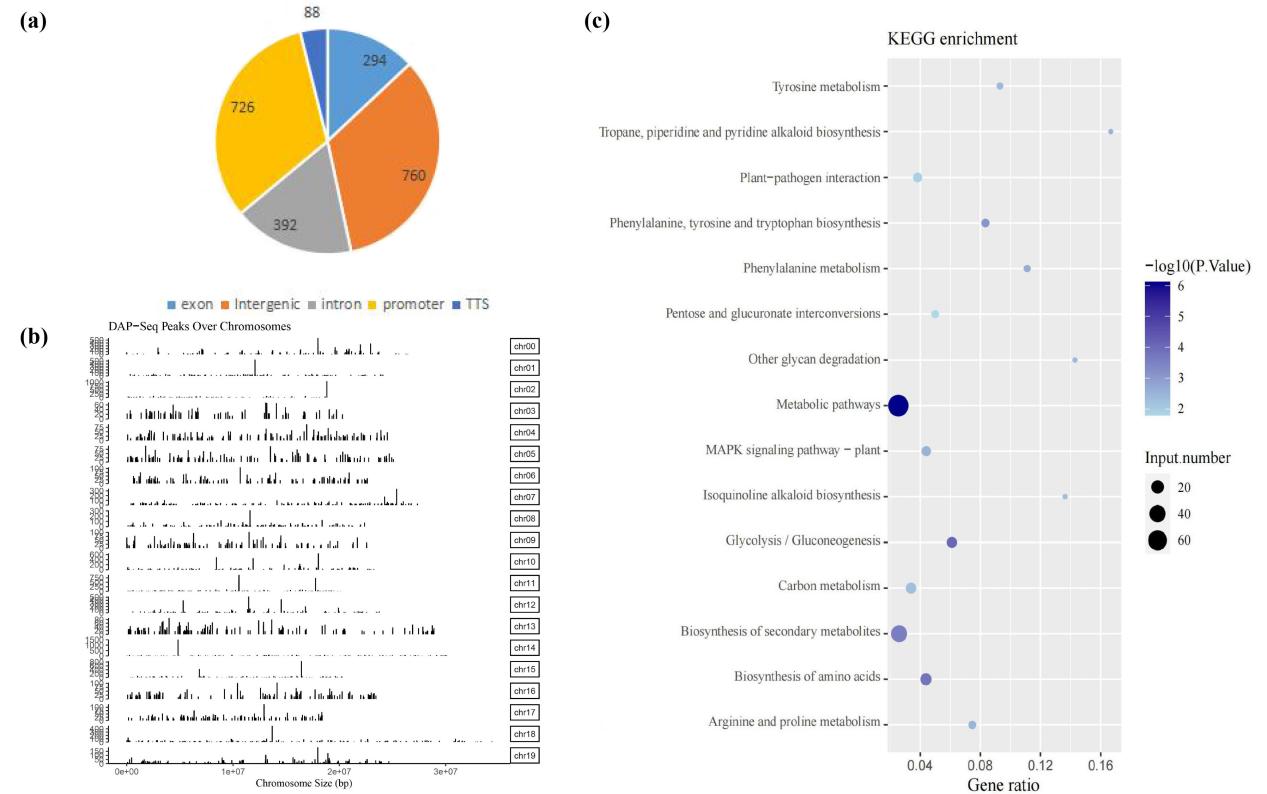


Figure. S3 Analysis of *VvMADS28* binding peak distribution by DAP-seq.

(a) Distribution of *VvMADS28* peaks in the grape genome based on localization of peak summits. The gene region is from 2 kb upstream of the transcription start site to 2 kb downstream of the transcription ending site. (b) Distribution of peaks on grape chromosomes. (c) KEGG analysis of *VvMADS28* binding site related genes.


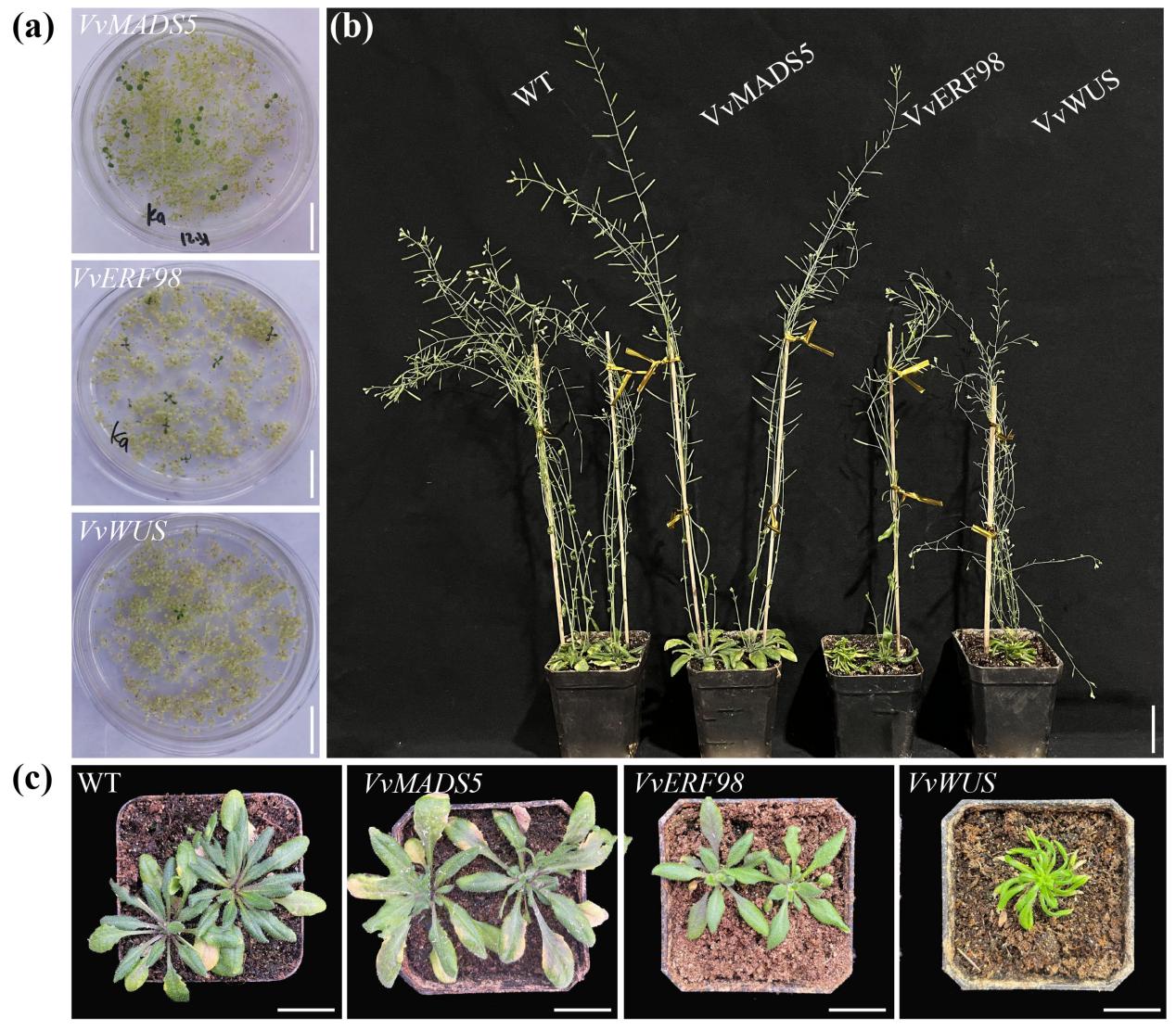


Figure. S4 Genetic transformation of *VvERF98*, *VvMADS5* and *VvWUS* in *Arabidopsis thaliana* and phenotypic observation.

(a) Positive lines were screened by MS medium plate containing antibiotics; Bar=2cm. (b) Phenotype observation of overexpression lines; Bar=2cm. (c) Morphological observation of leaf development; Bar=2cm.


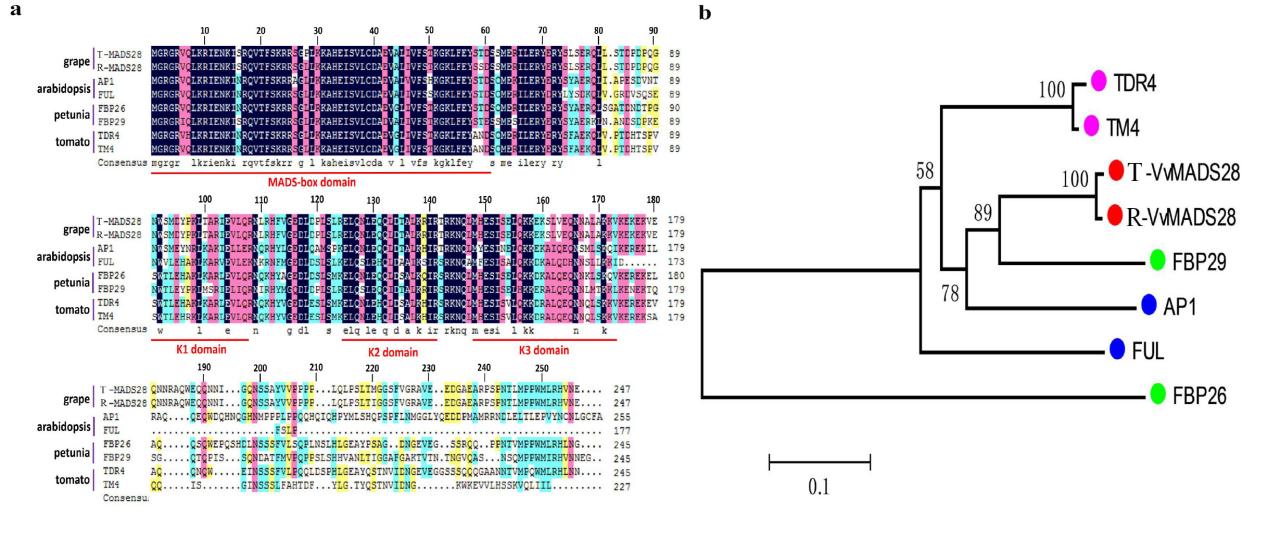
Figure. S5 Phylogenetic analysis of VvMADS28 and related proteins.

The scale bar represents 0.1 substitutions per site.


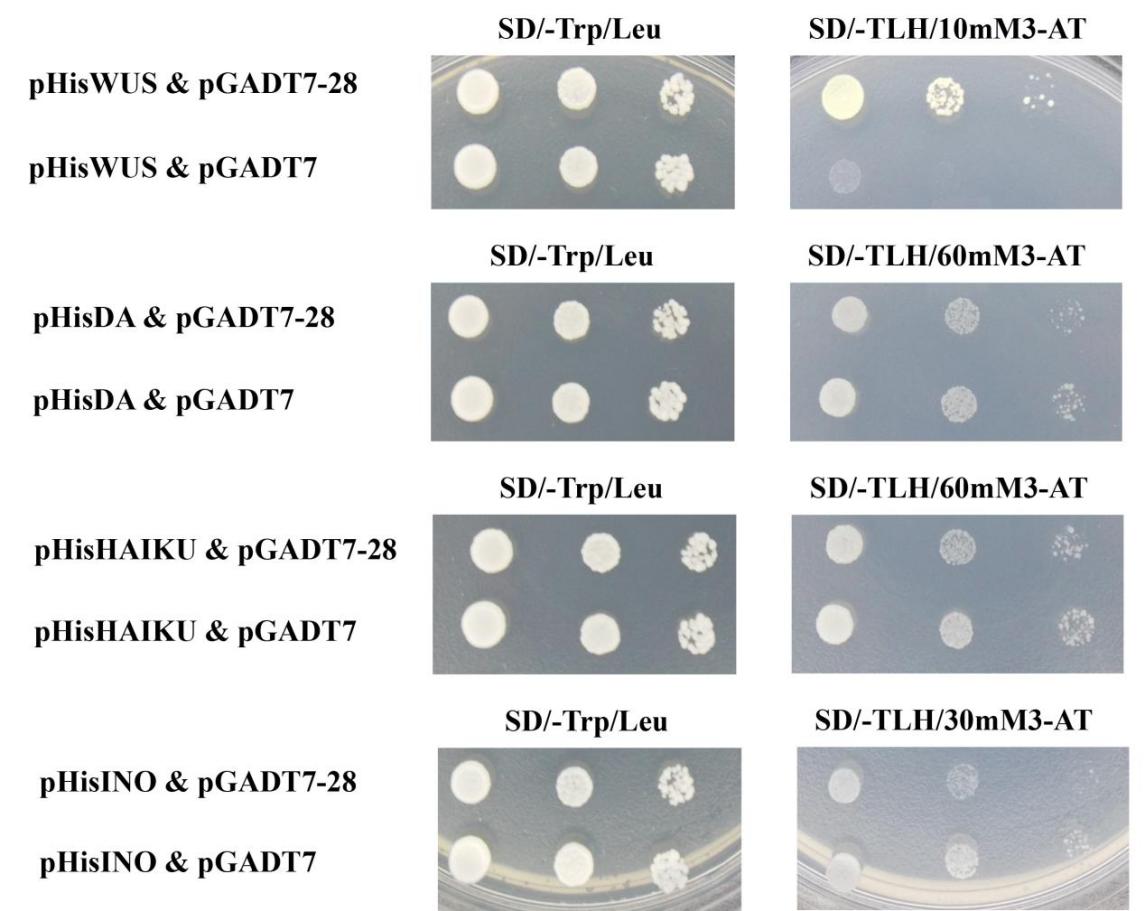


Figure. S6 Analyses of downstream target genes of *VvMADS28*.
